# Menstrual pattern and menstrual disorders among adolescents: an update of the Italian data

**DOI:** 10.1186/1824-7288-38-38

**Published:** 2012-08-14

**Authors:** Franco Rigon, Vincenzo De Sanctis, Sergio Bernasconi, Luigi Bianchin, Gianni Bona, Mauro Bozzola, Fabio Buzi, Giorgio Radetti, Luciano Tatò, Giorgio Tonini, Carlo De Sanctis, Egle Perissinotto

**Affiliations:** 1 Department of Paediatrics, University of Padua, Padua, Italy; 2 Private Accredited Hospital Quisisana, Paediatric and Adolescent Outpatients Clinic, Ferrara, Italy; 3 Department of Clinical and Experimental Medicine, University of Parma, Parma, Italy; 4 Child and Adolescent Psychiatry Unit, ULSS 16, Padua, Italy; 5 Division of Paediatrics, Department of Mother and Child Health, Azienda Ospedaliero-Universitaria Maggiore della Carità, Novara, Italy; 6 Internal Medicine and Therapeutics, Section of Childhood and Adolescence, University of Pavia, Foundation IRCCS San Matteo, Pavia, Italy; 7 Department of Paediatrics, "Carlo Poma" Hospital, Mantova, Italy; 8 Department of Paediatrics, Regional Hospital of Bolzano, Bolzano, Italy; 9 Department of Paediatrics, University of Verona, Verona, Italy; 10 Department of Paediatrics, Institute for Maternal and Child Health, IRCCS "Burlo Garofolo" and University of Trieste, Trieste, Italy; 11 Paediatric Department of Paediatrics, Koelliker Hospital, Turin, Italy; 12 Department of Cardiac, Thoracic and Vascular Sciences, University of Padua, Padua, Italy

**Keywords:** Menstrual pattern, Menstrual disorders, Menstrual cycle length, Bleeding length, Polymenorrhea, Oligomenorrhea, Dysmenorrhea, Adolescents

## Abstract

**Background:**

The most striking event in the whole process of female puberty is the onset of menstruation. To our knowledge, no large population-based studies have been performed on the topic of menstrual health among Italian adolescents in recent years.

The aims of this study were to produce up-to-date information on the menstrual pattern of Italian girls attending secondary school, and to estimate the prevalence of menstrual cycle abnormalities in this population.

**Methods:**

This was a cross-sectional study on a population-based sample of Italian adolescents aged 13–21 years attending secondary school. Only girls who had already started menstruating were requested to participate. Information was collected by means of a questionnaire that included items on the girls’ demographic details, anthropometrics, smoking and drinking habits, use of contraceptive pills, and socioeconomic status. The questions on the girls’ menstrual pattern concerned their age at menarche, duration of the most recent menstruation intervals (<21, 21–35, >35 days, variable), average days of bleeding (<4, 4–6, >6 days), and any menstrual problems and their frequency.

**Results:**

A total of 6,924 questionnaires were administered and 4,992 (71%) were returned. One hundred girls failed to report their date of birth, so 4,892 subjects were analyzed. The girls’ mean age was 17.1 years (SD ±1.4); their mean age at menarche was 12.4 (±1.3) years, median 12.4 years (95%CI 12.3–12.5).

In our sample population, 3.0% (95%CI 2.5%-3.4%) of the girls had menstruation intervals of less than 21 days, while it was more than 35 days in 3.4% (95%CI 2.9%-3.9%). About 9% of the girls (95%CI 7.7%-9.4%) said the length of their menstruation interval was currently irregular. Short bleeding periods (<4 days) were reported in 3.2% of the sample population (95%CI 2.7%-3.7%), long periods (>6 days) in 19% (95%CI 17.9%-20.1%). Menstruation-related abdominal pain was reported by about 56% of our sample. About 6.2% of the girls (95%CI 5.4%-7.0%) were suffering from dysmenorrhea.

**Conclusions:**

In conclusion, to the best of our knowledge, this is one of the largest studies on menstrual patterns and menstrual disorders among Italian adolescent girls. Adolescent girls referring persistent oligomenorrhoea, in first two years from menarche, had a higher risk for developing a persistent menstrual irregularity. They had longer bleeding periods (>6 days) and this has practical implications because it makes these adolescents potentially more susceptible to iron deficiency anemia. Clinicians need to identify menstrual abnormalities as early as possible in order to minimize their possible consequences and *sequelae*, and to promote proper health information.

We recommend that adolescents should be encouraged to chart their menstrual frequency and regularity prospectively from the menarche onwards.

## Introduction

Adolescence is the time of life between puberty and psychophysical maturity when crucial endocrinological, metabolic, somatic and psychological changes occur in girls. During this process, sequential phases mark the maturation of the complex endocrinological system that comprises the hypothalamus, pituitary gland, and ovary, and their interactions. Healthy reproductive function is the expected endpoint of this process [1-3].

The timing of this process is individual-specific, within a broad range of normality. The most frequent menstrual disorders are polymenorrhea, oligomenorrhea and dysmenorrhea [4-7]. Menstrual abnormalities are more common among younger girls, becoming less frequent as they grow older, 3–5 years after menarche [8-12]. Clinical evidence from the literature indicates that as of the third year after menarche the interval between bleeding periods is in the range of 21–34 days, with a flow lasting from 3 to 7 days and a mean menstrual blood loss of 35 ml (range 5–80 ml) [4-6]. Frequent anomalies outside normal references occasionally occur, or may become chronic, suggesting a shift from the normal endocrine-gynecological functional axis. Occasional deviations usually have temporary causes, such as psychological or physical stress, while chronic anomalies are much more likely to have pathological organic causes such as polycystic ovary, endometriosis, hypogonadism or cancer.

Population-specific reference data are useful to establish what is normal and acceptable, and what is not. Few population studies have been conducted in Italy on normal and dysfunctional characteristics of menstrual cycles. Since a knowledge of their variability is needed for patient education purposes and to guide clinicians’ investigations, treatment and follow-up, a cross-sectional school survey was conducted in 16 Italian cities (all over the country).

## Methods

### Sample and questionnaire

This was a cross-sectional study on a population-based sample of Italian adolescents aged 13–21 years attending secondary school. Information was collected by means of a questionnaire. A list of secondary schools was randomly selected in 16 Italian cities located all over the country (Brescia, Bolzano, Ferrara, Foggia, Lecce, Modena, Novara, Padova, Parma, Pavia, Reggio Emilia, Taranto, Torino, Trieste, Verona, Vicenza). All the schools agreed to take part in the study and a local investigator explained the aims of the survey and the questionnaire to the science teachers at each school, who relayed this information to their students and distributed the questionnaires. Only girls who had already started menstruating were requested to participate. Informed written consent was obtained from the students and their parents/guardians.

The girls were asked to complete an anonymous, self-administered questionnaire. Details on the sampling strategy used and on the questionnaire have been described in a previous publication [12].

The questionnaire included items on the girls’ demographic details, anthropometrics (weight, height), smoking and drinking habits (yes/no; frequency), use of contraceptive pills and reasons for their use (as a contraceptive, to regularize their menstrual cycle, or both), and socioeconomic status (Hollingshead index [13], where socioeconomic status is scored from 0 for the lowest social level to 14 for the highest).

The girls were asked to indicate their birth date (day, month, year) and, as accurately as possible, the date of their first menstrual bleeding (at least the month and the year); when the day was missing, the event was considered as it was happened at half month.

Age at interview was obtained as difference between interview and birth date divided by 365.25. Menarcheal age was computed as difference between birth and menarcheal date divided by 365.25. Age at interview and age at menarche were expressed as decimal year.

The questions on the girls’ menstrual pattern concerned their age at menarche, duration of the most recent menstruation intervals (<21, 21–35, >35 days, variable), average days of bleeding (<4, 4–6, >6 days), and any menstrual problems and their frequency. The impact of the menstrual cycle on the girls’ physical and psychological complaints was also investigated.

The following definitions were used to describe menstrual cycle disorders: polymenorrhea was defined as a menstruation interval lasting less than 21 days; oligomenorrhea as a menstruation interval of more than 35 days [14,15]; dysmenorrhea as abdominal pain severe enough to interfere with normal activities, or require medication. Abdominal pain was ranked on four levels (the last of which was termed as dysmenorrhea), as follows: no or mild/moderate abdominal pain; severe abdominal pain without any use of drugs, or sufficient to limit the girl’s activities; severe abdominal pain treated with drugs, and/or activity limitations during bleeding days; or severe abdominal pain treated with drugs and/or activity limitations before bleeding days [16,17].

### Statistical analysis

Descriptive statistics were obtained to describe the study population as a whole and divided into subgroups. Mean values and standard deviations (SD) were calculated for quantitative variables; median, first and third quartile were evaluated for age at menarche. Frequency distributions were calculated for qualitative variables. The prevalence of polymenorrhea, oligomenorrhea and dysmenorrhea was estimated with 95% confidence intervals (95%CI). Differences in proportions were analyzed using the chi square test or Fisher’s exact test, as appropriate. The chi square test for trend was used to assess the role of ordinal variables. Parametric and non-parametric one-way analyses of variance (ANOVA) were used to check differences between mean values of quantitative variables.

The linear trends of mean values for quantitative variables were estimated by applying simple linear regression models.

All statistical analyses were performed using SAS statistical software, rel. 9.1 (SAS Institute, Cary, NC, USA). The significance level was set at 0.05 and all tests were two-tailed.

## Results

A total of 6,924 questionnaires were administered and 4,992 (71%) were returned. The sample population only included Caucasian girls. One hundred girls failed to report their date of birth, so 4,892 subjects were analyzed. Only for analyses regarding the date at menarche the sample population sized at 3,783 (77%) with complete information.

The girls were a mean 17.1 years of age (SD ±1.4); their mean age at menarche was 12.4 (±1.3) years, median 12.4 years (first quartile 11.5 years; third quartile 13.2 years). They generally belonged to middle- to high-class families, with only 9% of them scoring lower than 7 on the social scale.

Table 1 shows the general characteristics and the age at menarche for the sample as a whole and for the various age groups. Anthropometric and lifestyle changes emerged with age, including a significant increase in the numbers of smokers (from 19% to 42%; p < 0.0001), drinkers (from 38% to 56%; p < 0.0001) and contraceptive pill users (from 2% to 25%; p < 0.0001) among the older groups, and a decrease in the prevalence of physical activity (from 58% to 39%; p < 0.0001). It is worth noting that the rising trend seen in the age of menarche with increasing age at the time of answering the questionnaire (from 12.1 to 12.6 years) was due to the study design. Given that only girls who had already their first menses were included, the older groups – unlike the youngest girls - also included girls who had experienced their menarche when more than 14 years old (the age at which they started secondary school). In line with the well-known association between a girl’s and her mother’s age at menarche, our findings confirmed the recognized tendency for girls to be younger at menarche than their mothers [12].

**Table 1:** General characteristics of a population-based sample of Italian secondary school girls

		**Age at data collection**							
	**Whole sample**	**<=14 years**	**15**	**16**	**17**	**18**	**19**	**> = 20**	**p for trend**
n	4892	301	501	1335	1433	952	258	107	
Weight (kg)	55.5 ± 7.6	53.3 ± 8.1	54.5 ± 7.8	55.4 ± 7.0	55.5 ± 7.6	55.7 ± 7.6	56.7 ± 8.3	56.8 ± 8.9	<0.0001
Height (cm)	165.5 ± 6.1	163.4 ± 5.8	165.1 ± 6.1	165.7 ± 5.9	165.6 ± 6.2	165.9 ± 6.1	165.8 ± 6.5	164.7 ± 6.7	<0.0001
BMI (kg/m^3^)	20.3 ± 2.5	20.0 ± 2.7	20.4 ± 2.6	20.2 ± 2.3	20.3 ± 2.5	20.2 ± 2.4	20.7 ± 2.8	21.0 ± 2.9	0.01
Family size (n°)	2.0 ± 0.7	1.9 ± 0.7	2.1 ± 0.7	1.9 ± 0.7	1.9 ± 0.7	1.9 ± 0.7	2.0 ± 0.7	2.0 ± 0.7	0.12
Smoking habit (%)	32.6	19.3	26.8	26.7	35.8	39.3	43.8	42.1	<0.0001
Alcohol drinking (%)	55.0	38.2	46.1	51.2	59.3	62.7	58.5	56.1	<0.0001
Physical activity (%)	50.0	57.8	57.9	56.2	50.0	39.4	36.8	39.3	<0.0001
Previously on pill (%)	22.0	12.3	21.4	15.3	21.8	28.6	38.8	40.2	<0.0001
Currently on pill (%)	12.6	2.3	5.6	7.6	12.9	20.2	29.1	25.2	<0.0001
* for contraception* (%)	8.7	1.0	3.6	4.9	9.0	13.5	21.7	21.5	<0.0001
* for cycle regularity* (%)	4.8	1.3	2.4	2.8	5.3	7.7	10.5	5.6	<0.0001
Age at menarche (years)	12.4 ± 1.3	12.1 ± 1.1	12.3 ± 1.3	12.3 ± 1.2	12.5 ± 1.3	12.5 ± 1.3	12.6 ± 1.5	12.6 ± 1.6	<0.0001
Mother’s age at menarche (years)	12.7 ± 1.6	12.6 ± 1.5	12.8 ± 1.7	12.6 ± 1.5	12.7 ± 1.6	12.8 ± 1.6	12.8 ± 1.8	12.9 ± 1.6	0.07

The following data describing the features of our sample’s menstrual pattern were obtained.

In our sample population, 3.0% (95%CI 2.5%-3.4%) of the girls had menstruation intervals of less than 21 days, while it was more than 35 days in 3.4% (95%CI 2.9%-3.9%). About 9% of the girls (95%CI 7.7%-9.4%) said the length of their menstruation interval was currently irregular, whereas about 72% reported it having done so in the past. Short bleeding periods (<4 days) were reported in 3.2% of the sample population (95%CI 2.7%-3.7%), long periods (>6 days) in 19% (95%CI 17.9%-20.1%).

When asked about any previous experience of polymenorrhea or oligomenorrhea, these conditions were reported by 34% and 51% of the girls, respectively.

**Figure 1:**
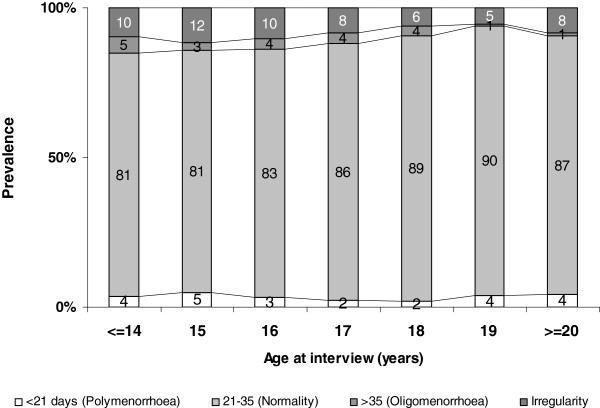
Distribution of duration of menstruation interval by age at data collection (p = 0.0002).

**Figure 2:**
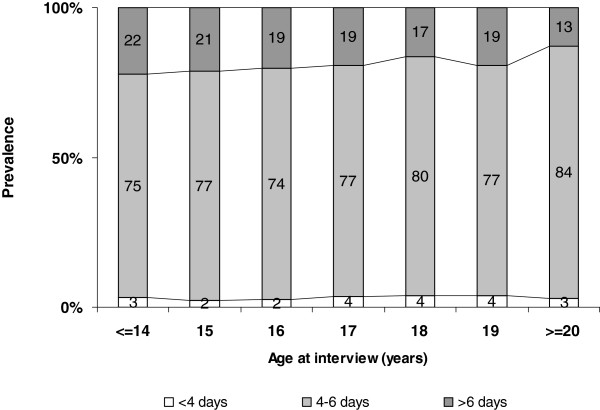
Distribution of duration of bleeding period by age at data collection (prevalence of >6 days, p for trend 0.008).

Figures 1 and 2 show the menstruation intervals and bleeding periods by age at interview: the proportion of girls with a regular cycle rose constantly from 81% at 14 years of age to 87% at 20 years old (p = 0.0002), while the prevalence of long bleeding periods (>6 days) dropped from 22% to about 13% (p for trend =0.009).

**Figure 3:**
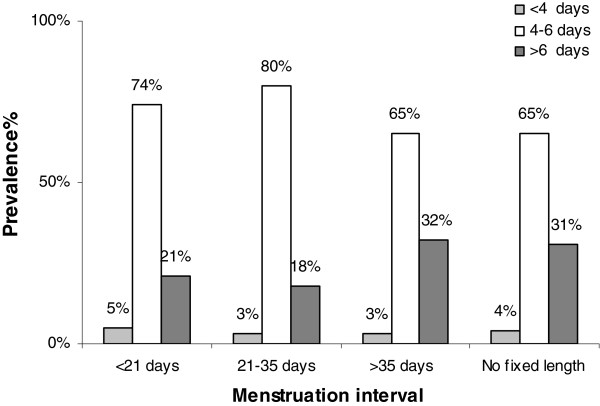
Distribution of length of bleeding period by length of menstruation interval (p < 0.0001).

Figure 3 shows the association between menstruation interval and bleeding period (p < 0.0001). Oligomenorrhea was related to longer bleeding periods (32%) and also a variable menstruation interval (31%).

**Figure 4:**
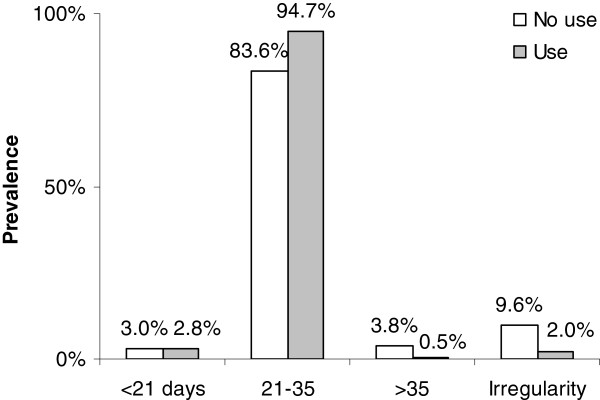
Distribution of length of menstruation interval by use of contraceptive pill (p = <0.0001).

As shown in Figure 4, our results indicate that menstruation interval was significantly more regular among girls using the pill (p < 0.0001).

Menstruation-related severe abdominal pain was reported by about 56% of our sample. Among those girls, 42% took pain medication and 11% of them experienced limitations in their normal activities. Among girls with severe pain, 10% satisfied the criteria for dysmenorrhea, while referring to the whole sample population the prevalence of dysmenorrhea was 6.2% (95%CI 5.4%-7.0%).

**Figure 5:**
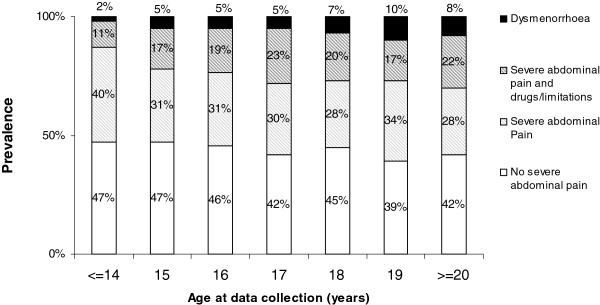
Prevalence of severe abdominal pain, from none to dysmenorrhea, by age at data collection (p for trend < 0.0001).

In the whole sample, the prevalence of dysmenorrhea (Figure 5) significantly increased with age, rising from about 2% in the youngest age group age to about 8% among the over 17 year-olds. It was not significantly associated with the length of the menstruation interval, but there was clearly a significant association with the length of the bleeding period, the prevalence being about 7% among subjects with bleeding periods <4 days or >6 days, as opposed to about 5% for those with periods lasting 4–6 days (p = 0.01).

## Discussion

The most striking event in the whole process of female puberty is the onset of menstruation.

The average age of menarche in Western European countries appears to have dropped over the past 150 years from over 16 to under 14 years old [18]. In Italy, the mean age at menarche was estimated at 12.4 ± 1.3 years [12,19].The declining age of puberty has been attributed to better living standards, e.g. adequate nutrition and health care [7,12].

The onset of menarche does not mean that the pituitary-gonadal axis (H-P-G) is fully developed and capable of regular function [20,21].

Although menstrual irregularities in adolescent girls are often attributed to an immature H-P-G axis [22], many adolescents with persistent menstrual abnormalities may raise concern of polycystic ovarian syndrome (PCOS) [8,9,23]. PCOS affects about 5% to 10% of women [24,25] in their reproductive life and can be associated with reproductive disorders, cardiovascular disease, type II diabetes mellitus and metabolic syndrome [26-28]. Because the clinical presentation of PCOS varies, it is important to have specific diagnostic criteria. In 2003, the ESHRE/ASRM Consensus introduced new phenotypes of PCOS requiring two of the following diagnoses of PCOS: chronic anovulation, biochemical and or clinical hyperandrogenemia and distinct sonographic appearance of ovaries [29]. The clinical significance of these phenotypes is controversial and the Androgen Excess Society position statement did not declare the inclusion of anovulatory non-hyperandrogenic women with ultrasonographic polycystic ovarian morphology in the PCOS cohort [30].

The schoolgirls included in our study had their first menstrual period at approximately the same age as their mothers, though there was a tendency for girls to be younger at menarche than their mothers. Similar results have been reported by Russo et. al [19] in a large population of Italian adolescents. The mean time lapse from B2 to B3 and B2 to menarche was 1.4 and 2.7 years, respectively [19]. These data further support for a genetic effect on age at menarche [1,2,12],

BMI, family size and birth order are other factors believed to influence age at menarche [31-34]. Although Gallo [33] found a mean age at menarche of 12.8 years for girls born in Northern Italy from large families as opposed to 12.6 for those from small families and Malina et al. [34] also reported a significant correlation between the age at menarche, family size and birth order, in our study the effect of family size was irrelevant.

By the mean age of 17.1 years, 3% of the subjects had cycles shorter than 21 days and in 3.4% they were longer than 35 days. A shorter than normal bleeding period (< 4 days) was reported by 3.2% of our sample population, and a long bleeding period (> 6 days) by 19% of the girls.

Dewhurst et al. [35] analyzed 368 menstrual periods and found that the flow lasted between 3 and 7 days in 88% of the cycles, with an average length of 5 days. In a larger series described by Widholm and Kantero [36], short periods of 2–3 days occurred in 8.8% of the girls during the first menstrual year, but this figure dropped to 3.7% by the fifth year.

It is important to remember that prolonged menstruation intervals and heavy menstrual bleeding warrant attention when these conditions are associated with anovulation or oligo-ovulation, hirsutism or moderate-severe acne [37-39]. Polycystic ovary syndrome (PCOS), Cushing’s disease, thyroid dysfunctions, premature ovarian failure, strong physical exercise, eating disorders, congenital adrenal hyperplasia, ovarian and adrenal tumors or prolactinomas are all examples of endocrine dysfunctions that can cause oligomenorrhea. Although some of these conditions are rare, others are more common and warrant careful evaluation and management, especially in cases with signs of androgen excess, irrespective of the subject’s menstrual or gynecological age [5,11,23,37,38].

Studies on female teenagers with heavy bleeding or hemorrhage have also identified coagulopathies in up to 20% of cases, the most common being von Willebrand disease, which occurs in as many as 15% of women [40,41].

The challenge for the physician is to distinguish between bleeding abnormalities secondary to anovulation and disorders requiring further investigation and a specific follow-up, as well as screening adolescents at risk for iron deficiencies [42-44].

Menstruation may be associated with various symptoms occurring before or during menstrual flow. A significant number of our students complained of dysmenorrhea, and this was more common among older girls with longer bleeding periods (16.7% among those whose bleeding period lasted <4 days, 24.1% for periods lasting 4–6 days, and 37.2% for periods lasting >6 days, p < 0.0001).

Our findings on the prevalence of dysmenorrheal only slightly differ from previous reports on a sample of 2,411 secondary school girls and 107 medical college students [7,39] where the prevalence of dysmenorrhea was ≈ 70% (6.3% severe, 30.4% moderate and 63.3% mild).

Although dysmenorrhea in adolescent and young adults is usually primary and is associated with normal ovulatory cycles, there is evidence that in approximately 10% of subjects with severe dysmenorrhea, pelvic or uterine anomalies may be found. Endometriosis is a cause of chronic pelvic pain and its presence is suggested by bowel or bladder symptoms or intercourse related pain [5,7].

Our study also showed a significant increase over time in the proportion of girls drinking alcohol, smoking cigarettes, or using oral contraceptives (OC) to prevent pregnancy, whilst their physical activity decreased with age. Similar results have been reported in several Italian reports referring to the same period [45-47]. The significant rising trend in the proportion of adolescent girls who smoke at a younger and younger age, combined with the increasing use of OC and reduction in physical activity paint a picture that raises a public health concern because it may increase these girls’ future morbidity and mortality rates. Health education schemes should be implemented right from kindergarten through 12^th^ grade to reduce health-risk-related behavior [48-51].

Our study has some limitations that need to be addressed. The survey was cross-sectional in design and we could not validate the self-reported information about the girls’ menstrual cycles, though the large size of our sample probably sufficed to enable a robust analysis of the menstrual outcomes. Another limitation lies in that no information was obtained on any medical management of dysmenorrhea, though the adolescents reported in the questionnaire whether they took medication for menstrual pain control.

In conclusion, to the best of our knowledge, this is one of the largest studies on menstrual patterns and menstrual disorders among Italian adolescent girls. Our results are consistent with other studies and confirm recent findings on the physiological events involved in the maturation of the female reproductive system.

Adolescents whose menstrual cycles are consistently outside the normal range should be assessed for pathological conditions. No studies have specifically tackled the issue of the duration of the menstrual bleeding period in relation to the menstruation intervals. To the best of our knowledge and literature consultation we found, for the first time, that adolescent girls with oligomenorrhea had longer bleeding periods (>6 days) and this has practical implications because it makes these adolescents potentially more susceptible to iron deficiency anemia.

To sum up, clinicians need to identify menstrual abnormalities as early as possible in order to minimize their possible consequences and *sequelae*, and to promote proper health information. Health education programs for adolescents remain an important area to develop further for the purposes of prevention.

We recommend that adolescents should be encouraged to chart their menstrual frequency and regularity prospectively from the menarche onwards to focus their attention on the need to take care of their health relating to any menstrual problems.

## Competing interests

The authors have no competing interests to declare.

## Authors’ contributions

All authors were involved in the conception and design of the study. EP carried out data extraction and conducted statistical analyses. FR, VDS, SB, GB, LB,CDS, EP drafted the paper with contributions from the co-authors. FR,VDS, EP revised the manuscript. All authors have read and approved the final version of the manuscript.
